# Enhanced Ant Colony Optimization with Dynamic Mutation and Ad Hoc Initialization for Improving the Design of TSK-Type Fuzzy System

**DOI:** 10.1155/2018/9485478

**Published:** 2018-01-15

**Authors:** Chi-Chung Chen, Yi-Ting Liu

**Affiliations:** Department of Electrical Engineering, National Chiayi University, 300 Syuefu Road, Chiayi City 60004, Taiwan

## Abstract

This paper proposes an enhanced ant colony optimization with dynamic mutation and ad hoc initialization, ACODM-I, for improving the accuracy of Takagi-Sugeno-Kang- (TSK-) type fuzzy systems design. Instead of the generic initialization usually used in most population-based algorithms, ACODM-I proposes an ad hoc application-specific initialization for generating the initial ant solutions to improve the accuracy of fuzzy system design. The generated initial ant solutions are iteratively improved by a new approach incorporating the dynamic mutation into the existing continuous ACO (ACO_R_). The introduced dynamic mutation balances the exploration ability and convergence rate by providing more diverse search directions in the early stage of optimization process. Application examples of two zero-order TSK-type fuzzy systems for dynamic plant tracking control and one first-order TSK-type fuzzy system for the prediction of the chaotic time series have been simulated to validate the proposed algorithm. Performance comparisons with ACO_R_ and different advanced algorithms or neural-fuzzy models verify the superiority of the proposed algorithm. The effects on the design accuracy and convergence rate yielded by the proposed initialization and introduced dynamic mutation have also been discussed and verified in the simulations.

## 1. Introduction

In contrast to the Mamdani-type fuzzy systems having good interpretability, Takagi-Sugeno-Kang- (TSK-) type fuzzy systems usually are employed for the accuracy-oriented applications. Since each rule of a TSK-type fuzzy system has a crisp output and the aggregated system output is computed via weighted average, thus avoiding time-consuming and mathematically intractable defuzzification operation, the TSK-type fuzzy system is a popular candidate for sample-based fuzzy modeling [1]. However, because fuzzy modeling usually requires high precision, the TSK-type fuzzy systems usually are designed or refined by using the optimization techniques to satisfy the requirement.

The optimization of the TSK-type fuzzy system design is to find the antecedent parameters characterizing the fuzzy sets for the inputs and the consequent parameters for the output by minimizing or maximizing the objective function. One of the major categories of solving such optimization problem is the gradient-based search methods, in which the search direction is derived based on the gradient of the objective function with respect to the parameters. To calculate the gradient, the gradient-based method needs the training input-output data pair of the fuzzy system, which, however, usually is a difficulty for fuzzy control problems because the target fuzzy outputs responding to the inputs are not available in advance. Another traditional difficulty for the gradient-based method is that it is easily trapped in a local optimum when it is applied to the optimization problems having multiple peaks in the design space such as the design of fuzzy system.

To avoid the issues encountered in the gradient-based approaches, many population-based computational techniques such as genetic algorithm [2] and swarm intelligence algorithms [3] have been proposed for solving optimization problems. These computational techniques are derivative free, so they can easily be applied to any optimization problems including the fuzzy control problems and the problems with nondifferentiable objective function. Since the search direction in these techniques is stochastic, they are more likely to traverse across the highly nonlinear design space, thus avoiding to be trapped into a local optimum. Furthermore, these approaches evaluate many candidate solutions concurrently, so they have better chance to find the better solution.

Genetic algorithm was inspired by the process of natural selection and developed based on the principle of survival of fittest. Genetic algorithms use bioinspired crossover operation between two selected parent solutions to produce child solutions and the mutation operation for further exploiting the generated individual child solutions for improving the performance. Genetic fuzzy systems [4–8] are the evolutionary fuzzy systems designed by genetic algorithms. For example, GA was used to design neural-fuzzy system for temperature control in [5] and fuzzy controller for mobile robots in [7].

Particle swarm optimization (PSO) is one of swarm intelligence models. PSO was inspired by the social behavior of fish schooling and bird flocking [9]. In the PSO, each particle represents a candidate solution to a problem and flies in the hyperspace according to its flying velocity vector, which is stochastically determined by its previously personal best and swarm best experiences. In the end of PSO operation, the experienced swarm best solution is the finally obtained solution. To address the encountered issues and improve over the parent PSO [9], many advanced PSO variants [10–15] have been proposed, some of which were applied to design the TSK-type fuzzy systems [14, 15]. These algorithms include the PSO with time-varying acceleration coefficients (PSO-TVAC) [10], a self-organizing hierarchical particle swam optimizer with TVAC (HPSO-TVAC) [10], PSO with controllable random-exploration velocity (PSO-CREV) [11], enhanced PSO by incorporating a weighted particle [12], the hybrid of GA and PSO (HGAPSO) [13], two-phase swarm intelligence algorithm (TPSIA) [14], and ant and particle swarm cooperative optimization (APSCO) [15].

Another type of swarm intelligence model is ant colony optimization (ACO) [16–19]. The ACO technique was inspired by foraging behavior of the real ant colony and was proposed initially for solving discrete combinational optimization problems such as travelling salesman problem (TSP). The ACO algorithms have been successfully applied to optimize the fuzzy systems for mobile robot control [20, 21]. In those studies, in order to apply the discrete ACO for optimization, the parameters charactering the fuzzy controller were first discretized, thus sacrificing the precision.

To overcome the precision issue, some ant-related algorithms for the optimization problems with real-value parameters have been proposed [22–24]. Among these algorithms, this paper focuses the interest on ACO_R_ [24] because it is most related to the discrete ACO and has achieved good performances on continuous optimization of the benchmark functions as demonstrated in [24]. ACO_R_ thus is advantageous on the application problems requiring high precision such as the design of fuzzy controllers for dynamic systems because their inputs and outputs are usually continuous cases. Since the proposal of ACO_R_, some variants have been proposed and applied to design fuzzy systems for accuracy-oriented problems [25–28]. In [25], a modified continuous ACO algorithm (RCACO) was proposed for the design of fuzzy-rule-based systems in order to achieve considerable learning accuracy. The paper [26] proposed a cooperative continuous ACO (CCACO) with multiple colonies of populations, each colony of which is only responsible for optimizing a single fuzzy rule. Although the simulation results demonstrated that the performance of multicolony based CCACO is better than that of the single-colony RCACO, the computation of CCACO is much more complex than that of RCACO. Inspired by the cognitive psychology concepts, the study in [27] proposed an assimilation-accommodation mixed continuous ant colony optimization (ACACO) for the designs of feed-forward fuzzy systems. In addition, the elite-guided continuous ACO (ECACO) was proposed to design recurrent fuzzy systems [28], which was claimed to be the first application of continuous ACO on recurrent fuzzy system design. Although those variants indeed helped accomplish the accurate design of fuzzy systems, there is still possible room for further improvement, especially on the balance between the exploration and convergence rate of solutions. This paper introduces the dynamic mutation [29] into ACO_R_ to enhance such balance.

For population-based algorithms such as PSO and ACO, another major issue is population initialization [30–34]. Starting with a population of initial solutions, the population-based algorithm improves the solutions iteratively in order to find the better solution. Therefore, the population initialization affects the performance. In general, however, the initial population solutions are generated randomly because of no a priori information. The work in [31] suggested use of Sobol sequence generator for generating initial PSO particles because the generated initial solutions are uniformly distributed into the search space. The study in [32] reported that the initialization using the nonlinear simplex method (NSM) helped improve the convergence rate and success rate of PSO. By using the generators of centroidal Voronoi tessellations (CVT) as the starting point, as suggested in [33], the initial solutions can be more evenly distributed throughout the high-dimensional problem space and improve PSO performance. The study in [34] proposed a center-based sampling for the initialization of population-based algorithms. By simulations, that paper showed that the points in center region have higher chances to be closer to an unknown solution and thus suggested the center region is a promising region for the initialization. However, those initialization approaches [31–34] did not take into account application-specific information. Based on [34] and the observations of some accuracy-oriented fuzzy controller designs, this paper proposes an ad hoc population initialization method for initial ant solutions to improve the design accuracy.

This paper mainly contributes to propose an enhanced continuous ACO algorithm incorporating dynamic mutations and ad hoc initialization, ACODM-I, for the design of TSK-type fuzzy systems. ACODM-I can be regarded as a population-based evolutionary algorithm. Instead of random generation, the proposed population initialization in ACODM-I takes the observations of well-performed fuzzy systems into account. The introduced dynamic mutation in ACODM-I initially provides more diverse search directions for exploring solutions to avoid being trapped into a local optimum in the early stage. The performance superiority of ACODM-I to the parent ACO_R_ and different advanced algorithms or neural-fuzzy models is verified by the simulation results of TSK-type fuzzy systems for the problems of tracking control and chaotic time series predication. Furthermore, the effects on the convergence rate and design accuracy yielded by the proposed initialization and introduced dynamic mutation are verified by the simulation results.

This paper is organized as follows. The next section describes the zero-order and first-order TSK-type fuzzy systems.  Section 3 introduces basic concepts of discrete ACO and ACO_R_ and proposes ACODM-I for TSK-type fuzzy system design.  Section 4 presents the simulation results of TSK-type fuzzy systems designed by ACODM-I for the tracking control of dynamic plant and the prediction of chaotic time series.  Section 4 also compares the ACODM-I performance with the ones of ACO_R_ with different values of parameter and advanced algorithms or models. Furthermore, the effects of the proposed initialization and dynamic mutation are also discussed and verified in Section 4. Ultimately, conclusion remarks are given in Section 5.

## 2. TSK-Type Fuzzy System

In contrast to Madami-type model having good interpretability, TSK-type fuzzy systems focus on the model accuracy. The main difference is on the consequent part of fuzzy rules. In a TSK-type fuzzy system, the consequence of fuzzy rule is defined as the linear combination of the fuzzy inputs. The *i*th fuzzy rule of a TSK-type fuzzy system is described as follows:

(1) $$\begin{array}{c} \text{Rule }i\text{: }If\;x_{1}\left(k\right)\;\;is\;\;A_{i1},\ldots ,x_{n}\left(k\right)\;\;is\;\;A_{in},\\ Then\;y\left(k\right)\;\;is\;\;f_{i}\left(x_{1},x_{2},\ldots ,x_{n}\right), \end{array}$$

where *k* is the time step, *x*_1_, *x*_2_,…, *x*_*n*_ are the input variables, and *y* is the output variable of the fuzzy system. In the antecedent part of fuzzy rule *i*, *A*_*ij*_ is a fuzzy set for the input *x*_*j*_ and is characterized by a membership function. If the function *f*_*i*_(*x*_1_, *x*_2_,…, *x*_*n*_) in the consequent part is a constant

(2) $$\begin{array}{c} f_{i}\left(\;x_{1},x_{2},\ldots ,x_{n}\;\right)=a_{i}, \end{array}$$

the system is denoted as a zero-order TSK-type fuzzy system. If the function *f*_*i*_(*x*_1_, *x*_2_,…, *x*_*n*_) is defined by

(3) $$\begin{array}{c} f_{i}\left(\;x_{1},x_{2},\ldots ,x_{n}\;\right)=a_{i0}+\overset{n}{\underset{j=1}{\sum }}a_{ij}x_{j}, \end{array}$$

it is a first-order TSK-type fuzzy system. In this study, as widely used in fuzzy system designs [15–17, 27–30], Gaussian function is selected to characterize the fuzzy set *A*_*ij*_ and is defined by

(4) $$\begin{array}{c} M_{ij}\left(\;x_{j}\;\right)=\exp\left\{\;-{\left(\;\frac{x_{j}-m_{ij}}{b_{ij}}\;\right)}^{2}\;\right\}, \end{array}$$

where *m*_*ij*_ and *b*_*ij*_ are the center and the width of fuzzy set *A*_*ij*_, respectively. For a Gaussian function, the parameters *m*_*ij*_ and *b*_*ij*_ are* independent *variables and its function output corresponding to any input value is never zero, which will make the design task easier.

For a TSK-type fuzzy system consisting of *R* rules, the output of the fuzzy system through the inference engine is calculated by

(5) y=∑i=1Rϕix→·fix→∑i=1Rϕix→,ϕix→=∏j=1nexp−xj−mijbij2,

where $\phi_{i}\left(\vec{x}\right)$ is the firing strength of rule *i* excited by a given input dataset $\vec{x}=\left(x_{1},x_{2},\ldots ,x_{n}\right)$. Thus, to construct an *R*-rule TSK-type fuzzy system with *n* input variables, all decision variables represented by

(6) s→=m11,b11,…,m1n,b1n,a1,m21,b21,…,m2n,b2n,a2,…,mR1,bR1,…,mRn,bRn,aR≡s1,s2,…,sD,

for a zero-order TSK-type fuzzy system, or

(7) s→=m11,b11,…,m1n,b1n,a10,a11,…,a1n,m21,b21,…,m2n,b2n,a20,a21,…,a2n,mR1,bR1,…,mRn,bRn,aR0,aR1,…,aRn≡s1,s2,…,sD,

for a first-order TSK-type fuzzy system, are to be determined. However, such design task of a TSK-type fuzzy system can be treated as an optimization problem that finds the free parameters represented in (6) or (7) such that the task-dependent objective function is optimized. Following this transformation, the optimization algorithms can be used to solve such optimization problem for accomplishing the design of TSK-type fuzzy system.

## 3. Proposed ACODM-I for Fuzzy System Design

This section presents the proposed enhanced continuous ACO with dynamic mutation and ad hoc initialization (ACODM-I) for the design of TSK-type fuzzy systems. Since the proposed ACODM-I is inspired and related to the ACO framework, this section first reviewed the basic concept of the discrete ACO and ACO_R_. The simulation results optimized by the ACO_R_ will also be presented as the benchmark for comparison in Section 4.

### 3.1. Basic Concept of Discrete Ant Colony Optimization

The discrete ACO algorithms [16–19] were inspired and developed by the behavior of real ant colonies and now are largely employed to find the solution of discrete combinational optimization problems (COP). When ants foraged for food, they deposited the pheromone on the trail to guide other ants. Since the pheromone will evaporate with time, the path with a higher pheromone level is the most possibly a shorter one to the food source. Ant System (AS) [16] is the first discrete ACO algorithm and proposed to solve the travelling salesman problem (TSP). In Ant System for TSP, *P*_*ij*_^*k*^(*t*) represents the probability that ant* k* situated at city* i* at the time* t* chooses to visit city* j* and is defined by

(8) $$\begin{array}{c} P_{ij}^{k}\left(\;t\;\right)=\left\{\begin{array}{cc} \frac{{\left(\tau_{ij}\left(t\right)\right)}^{\alpha }{\left(\eta_{ij}\right)}^{\beta }}{\sum_{m\in V_{i}^{k}}{\left(\tau_{il}\left(t\right)\right)}^{\alpha }{\left(\eta_{il}\right)}^{\beta }} & if\;\;j\in V_{i}^{k},\\ 0 & otherwise, \end{array}\right. \end{array}$$

where *τ*_*ij*_(*t*) is the pheromone trail on the link between cities *i* and *j*, *η*_*ij*_ is the corresponding a priori heuristic information of the link, and *V*_*i*_^*k*^ is the set of allowed neighborhood cities ant *k* can move from city *i*. The relative significance of the pheromone trail and heuristic information is determined by the values of *α* and *β*. Each ant is assumed to visit each city only once, and it will construct a feasible solution to TSP after it visited all cities. After all ants accomplished their visits, all feasible solutions are gathered to update the pheromone levels for the next ant cycle. The discrete ACO algorithm repeats such procedure to find the shorter path. Since that proposal, some variants of discrete ACO algorithms were proposed [17, 18]. In addition, by discretizing the parameters in fuzzy systems, the applications of the discrete ACO to optimize the fuzzy controllers for mobile robots have been demonstrated [20, 21].

### 3.2. Basic Concept of ACO_R_

**Figure 1:**
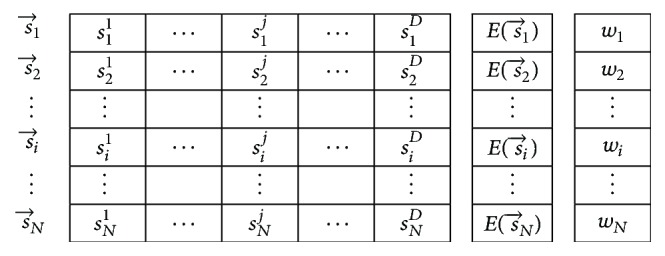
The solutions archive in ACO_R_, where the weights *w*_1_ ≥ *w*_2_ ≥ ⋯≥*w*_*N*_ and the objective values $E\left({\vec{s}}_{1}\right)\leq E\left({\vec{s}}_{2}\right)\leq \cdots \leq E\left({\vec{s}}_{N}\right)$ [24].

Among many proposed continuous ACO algorithms, the ACO_R_ in [24] is one of the most promising algorithms and is most related to the original discrete ACO. In [24], ACO_R_ has demonstrated good performances for continuous optimization of the benchmark functions. The basic concept of ACO_R_ is to extend the discrete probability distributions (8) utilized in discrete ACO to continuous Gaussian probability density functions (PDFs). In ACO_R_, these Gaussian PDFs are derived from a maintained solution archive as shown in Figure 1, and the sampled values from the chosen PDFs are gathered to construct new solutions.

In Figure 1, a feasible solution (ant path) to the optimization problem is represented by a row vector ${\vec{s}}_{i}$ in the archive, and its quality $E\left({\vec{s}}_{i}\right)$ is measured by the value of the predefined objective function. All feasible solutions are sorted, ranked from the best to the worst, and maintained in the fixed-size archive table. By doing this, the solution ${\vec{s}}_{l}$ thus has rank *l*. Then the rank of each ant solution in the archive determines its probability of being chosen to follow in the next ant cycle. The operation of ACO_R_ operation is detailed as follows.

ACO_R_ usually initializes all* N* solutions in the archive, each of which is a* D*-dimensional row vector $\vec{s}=[s^{1},s^{2},\ldots ,s^{D}]$, by generating random numbers within the range of search space. All initialized solutions then are evaluated, sorted according to the evaluation values, and ranked in the solution archive. Each solution ${\vec{s}}_{l}$ of the rank* l* in the sorted archive is assigned with a weight *w*_*l*_:

(9) $$\begin{array}{c} w_{l}=\frac{1}{qN\sqrt{2\pi }}\exp\left\{\;-\frac{{\left(l-1\right)}^{2}}{2q^{2}N^{2}}\;\right\}, \end{array}$$

where *q* is a parameter of the ACO_R_. To generate a new candidate solution, the ACO_R_ firstly chooses one leading solution ${\vec{s}}_{l}$ among *N* solutions in the archive according to the probability distribution

(10) $$\begin{array}{c} p_{l}=\frac{w_{l}}{\sum_{m=1}^{N}w_{m}},\;m=1,2,\ldots ,N. \end{array}$$

It indicates clearly from (10) that the better-ranked solution has higher probability being chosen as the leading solution and the value of* q* in (9) controls the tendency for exploring the archive solutions. Once a leading solution ${\vec{s}}_{l}$ is chosen, a new candidate solution is constructed by sampling the derived Gaussian PDF *g*_*l*_^*j*^(*s*; *μ*_*l*_^*j*^, *σ*_*l*_^*j*^) in a sequence of *j* = 1,2,…, *D* with the mean *μ*_*l*_^*j*^ = *s*_*l*_^*j*^ and the standard deviation *σ*_*l*_^*j*^

(11) gljs;μlj,σlj=1σlj2πexp⁡−s−μlj22σlj2,σlj=ε∑m=1Nsmj−sljN−1,

where the pheromone evaporation rate *ε* is a positive parameter. A higher value of *ε* provides more exploration in search thus converging slower while a lower value of *ε* provides more exploitation in search, thus converging faster. By repeating such process *L* times, *L* new candidate solutions are generated. Then these *L* new candidate solutions are evaluated. Together with the original *N* solutions in the previous ant cycle, the total (*N* + *L*) solutions are sorted again. The ACO_R_ only reserved the *N*-top-performed solutions for next ant cycle. The ACO_R_ repeats such ant cycle until the termination condition is satisfied.

### 3.3. ACO_R_ Using Dynamic Mutation and Ad Hoc Initialization (ACODM-I)

#### 3.3.1. ACO_R_ with Dynamic Mutation (ACODM)

In ACO_R_, the value of *q* in (9) controls the exploration ability thus affecting convergence rate of the algorithm. When the value of *q* is small, ACO_R_ strongly prefers the best-ranked solutions as the leading solution, which focuses on exploiting the best-ranked solutions locally. This will increase the convergence rate, but the chance of convergence result being trapped into the local optimum is also increased. When *q* value is large, the probability for each solution being chosen as the leading solution becomes nearly uniform. This can enhance the exploration ability for possibly obtaining globally better solution, but the convergence rate will be decreased.

In order to avoid or lessen this issue, this paper introduces mutation technique into the original ACO_R_ to balance the exploration ability and the convergence rate. In addition to the Gaussian sampling technique, the introduced mutation provides another option for changing (generating) a newly constructed solution component by “jumping” to the neighboring of the other archive solutions. Since the probability for the mutation is not fixed, which will be clearly seen, this modified algorithm is named as ACO_R_ with dynamic mutation, ACODM, and its operation is detailed as follows.

In ACODM, without loss of generation, the range of each decision variable to be identified is assumed in the interval [0,1]. If a leading solution ${\vec{s}}_{l}$ is chosen, the value of the* d*th component of a new candidate solution ${\vec{s}}_{i}$ is generated.

If rand_*i*_^*d*^ > *p*^*d*^(*t*),

(12) $$\begin{array}{c} s_{i}^{d}\left(\;t+1\;\right)=Sampl\left(\;g_{l}^{d}\left(\;s;s_{l}^{d}\left(\;t\;\right),\sigma_{l}^{d}\left(\;t\;\right)\;\right)\;\right), \end{array}$$

else

(13) sidt+1=srdt+srdt·U−0.1,0.1,if  srdt≤0.5sidt+1=srdt+1−srdt·U−0.1,0.1if  srdt>0.5.

In (12), the Sampl(*g*_*l*_^*d*^(*s*; *s*_*l*_^*d*^(*t*), *σ*_*l*_^*d*^(*t*))) denotes the sampled value from a Gaussian PDF *g*_*l*_^*d*^(*s*; *s*_*l*_^*d*^(*t*), *σ*_*l*_^*d*^(*t*)) with the mean *s*_*l*_^*d*^(*t*) and the standard deviation *σ*_*l*_^*d*^(*t*). The ranges of the uniform random numbers rand_*i*_^*d*^ and *U*[−0.1,0.1] are in [0,1] and [−0.1,0.1], respectively. The index *r* in (13) is a uniform random integer number in [1, *N*]. Finally, the mutation probability *p*^*d*^(*t*) for the *d*th component is set to be linearly proportional to the biased standard deviation *σ*^*d*^(*t*) among all ants and is calculated by

(14) $$\begin{array}{c} p^{d}\left(\;t\;\right)=h\cdot \sigma^{d}\left(\;t\;\right)=h\cdot {\left(\;\sum_{i=1}^{N}\frac{{\left(s_{i}^{d}\left(t\right)-\bar{s^{d}}\left(t\right)\right)}^{2}}{N}\;\right)}^{1/2}, \end{array}$$

where *h* is an adjustable parameter determining the mutation probability and $\bar{s^{d}}(t)$ is the average value among all the* d*th components in the ant population.

In ACODM, in addition to the exploitation using Gaussian sampling in (12), the dynamic mutation in (13) can explore more diverse search solutions to avoid being trapped into a local optima in the early stage of the optimization process. Moreover, the mutation probability for each solution component in ACODM depends on the convergence status of the ant population, so its value is not fixed but dynamic and is not the same for each solution component. In (14), a higher value of* h *suggests a possibly larger probability for dynamic mutation by directly jumping to the neighboring of the other archive solutions. Therefore, the higher the value of* h *is, the lower the convergence rate is. Finally, the change of value for the mutation of ACODM, as presented in (13), is dynamic in contrary to the fixed change used in the mutation operation of general genetic algorithms. Equation (13) indicates the points around the center region have larger possible deviation than those points close to the boundary when they are chosen as the neighbors for learning. The initial motivation for this thought is to hope the central points can wander and explore in more diverse search directions in the early stage of optimization process.

The ACODM algorithm repeats the selection of leading solution using (10) followed by the Gaussian sampling in (12) or dynamic mutation (13) to generate* L* new candidate solutions. These* L* new candidate solutions are evaluated and sorted together with the* N* solutions in the previous ant cycle. Similarly, only the* N*-top-best solutions are reserved for next ant cycle. ACODM repeats such ant cycle to find the better solution until the termination condition is met.

#### 3.3.2. Ad Hoc Population Initialization for Fuzzy System Design

The initialization of the ant solutions in ACO_R_ is another issue, which is also a major problem for all other population-based algorithms [30–35]. Starting with a population of initial ant solutions, ACO_R_ improves the solutions iteratively to find the better solution. Therefore, the initialization of ant solutions in ACO_R_ affects the performance. In general, good initialization can help achieve better optimum while bad initialization usually ends on a poor local optimum. However, because no a priori information is available, in general the initial ant solutions are generated randomly. As mentioned in Section 1, some advanced random initialization approaches over the multiple-dimensional search space were proposed [31–34]. However, those initialization approaches did not take into account application-specific information, and thus they are regarded as generic initialization.

The study in [34] proposed a center-based sampling for the initialization of population-based algorithms. By simulations, that paper showed that the points in [0.2,0.8] in the search space [0,1] have higher chances to be closer to an unknown solution, and thus the center region is a promising region for the initialization. In addition, in our observation of fuzzy systems, especially accuracy-oriented fuzzy controllers, one of the membership functions for each input in its antecedent part is usually designed at the location around the input value arising more frequently or most concerned, which is usually at the center of the search range. Therefore, this paper proposes an ad hoc central initialization range [0.45,0.55] for initializing the parameters *m*_*ij*_ and *b*_*ij*_ in the antecedent part of fuzzy rule for the fuzzy system designs. The rest of free parameters *a*_*ij*_ in the consequent part of a TSK-type fuzzy system are initially generated uniformly in the search space because of no a priori information.

If ACODM generates the initial ant solutions using the proposed initialization method, the resultant algorithm is denoted by ACODM-I. For clarity, the pseudocode of the ACODM-I algorithm is shown in Algorithm 1.

## 4. Simulations

Three application examples of the designs of TSK-type fuzzy systems are demonstrated in this section to validate the proposed algorithm. Two zero-order TSK-type fuzzy systems are designed for nonlinear dynamic plant control, and one first-order TSK-type fuzzy system is optimized for the prediction of the chaotic time series. In the simulations, the population size* N* is 20 and the number of newly generated temporary solutions* L* is 20. The value of *ε* is 0.85 as used in [24, 25]. The value of $h=\sqrt{12}$ is set to have the mutation probability of 1.0 when the decision variable in each dimension is uniformly distributed among all ants. The performances of fuzzy controllers and fuzzy predictor optimized by ACODM-I are validated and compared with those by ACO_R_ with different values of* q*. In the simulations for ACO_R_, each initial parameter value in the fuzzy systems was generated randomly and uniformly within its search space. In addition, the resulting ACODM-I performance is also compared with the reported results of some advanced population-based algorithms or neural-fuzzy models on the same problem. The advantage on the optimization accuracy yielded by the proposed initialization and dynamic mutation are also discussed through simulation results. The personal computer for conducting all simulations possesses an Intel Core i5 2.8 GHz dual-core-processor and runs on Windows 7.

**Example 1 .**

As the first example, a zero-order TSK fuzzy system is designed to control the nonlinear plant as taken in [14, 25] and described by

(15) $$\begin{array}{c} y\left(\;k+1\;\right)=\frac{y\left(k\right)}{1+y^{2}\left(k\right)}+u^{3}\left(\;k\;\right). \end{array}$$

The initial state *y*(0) of the system is assumed to zero and −1 ≤ *u*(*k*) ≤ 1 is the control input of the plant. The objective for the fuzzy system (controller) to be optimized is to control the plant output to track the reference trajectory *y*_*d*_(*k*) as given by

(16) $$\begin{array}{c} y_{d}\left(\;k\;\right)=\sin\left(\;\frac{\pi k}{50}\;\right)\cos\left(\;\frac{\pi k}{30}\;\right),\;1\leq k\leq 250. \end{array}$$

In this example, the fuzzy controller is fed with two signals: the current plant output *y*(*k*) and the target output *y*_*d*_(*k* + 1). As the response to these two inputs, the produced output *u*(*k*) of the fuzzy system is used to control the nonlinear plant (15). For a designed fuzzy controller, its performance is evaluated by the root mean square error (RMSE) between the plant output and the reference trajectory over the 250 time steps and is calculated by

(17) $$\begin{array}{c} RMSE={\left(\;\sum_{k=1}^{250}\frac{{\left(y_{d}\left(k\right)-y\left(k\right)\right)}^{2}}{250}\;\right)}^{1/2}. \end{array}$$

**Figure 2:**
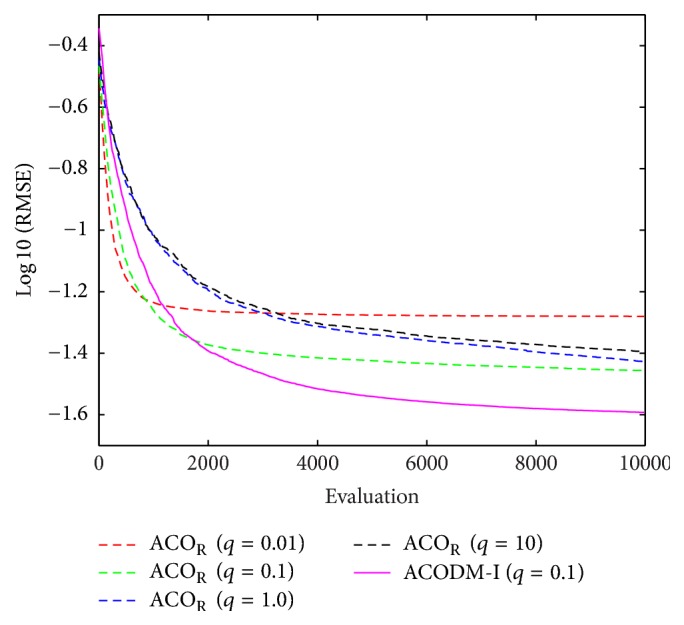
The average best-so-far RMSE at each performance evaluation for the evolutionary fuzzy controllers optimized by ACODM-I and ACO_R_ in Example 1.

The fuzzy controller in this example consists of five fuzzy rules in order to compare with other algorithms later. Then the values of the free parameters *m*_*ij*_ ∈ [−1,1], *b*_*ij*_ ∈ [0,1], and *a*_*i*_ ∈ [−1,1] for constructing a fuzzy system are searched using ACODM-I such that the error in (17) is minimized. For each single run of optimization process, 10000 evaluations were performed to conclude a solution. Over 50 runs of simulations, the average best-so-far RMSE at each performance evaluation of ACODM-I is shown in Figure 2. The learning results of the parent ACO_R_ with different values of *q* are also shown in Figure 2 for comparison. The learned statistical numerical results of RMSE errors are shown in Table 1. The results show that the average RMSE, minimum RMSE, and maximum RMSE of ACODM-I are smaller than the ones of ACO_R_ algorithms with different values of *q*.

The previous study [25] reported the performance results of some advance population-based evolutionary algorithms when they were applied to the same fuzzy control problem. These algorithms include a hierarchical PSO-TVAC (HPSO-TVAC) [10], a PSO with controllable random-exploration velocity PSO (PSO-CREV) [11], a hybrid of GA and PSO (HGAPSO) [13], a two-phase swarm intelligence algorithm (TPSIA) using discrete ACO in the first phase and PSO in the second phase [14], and fuzzy-rule-based continuous ant colony optimization (RCACO) [25]. The operations of these algorithms and their settings of parameters used on this control problem were detailed in [14, 25]. The fuzzy system optimized by each of these algorithms was also composed of five rules to ensure the same number of free parameters as that by ACODM-I. Moreover, for each single run, each of these algorithms also performed the same number of evaluations as that by ACODM-I.  Table 2 presents the reported performance results of these algorithms. The results show that ACODM-I achieved the smaller average error than all other algorithms in comparison.

**Figure 3:**
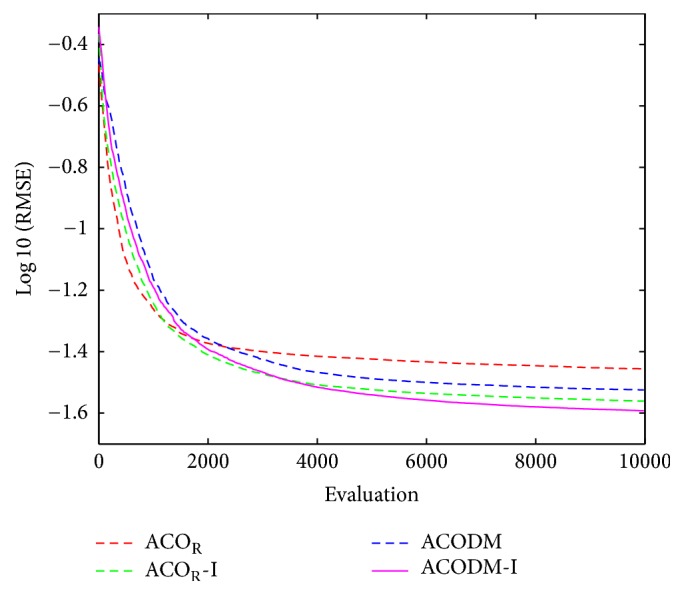
The average best-so-far RMSE at each performance evaluation for the evolutionary fuzzy controllers optimized by ACO_R_, ACODM, ACO_R_-I, and ACODM-I in Example 1.

**Figure 4:**
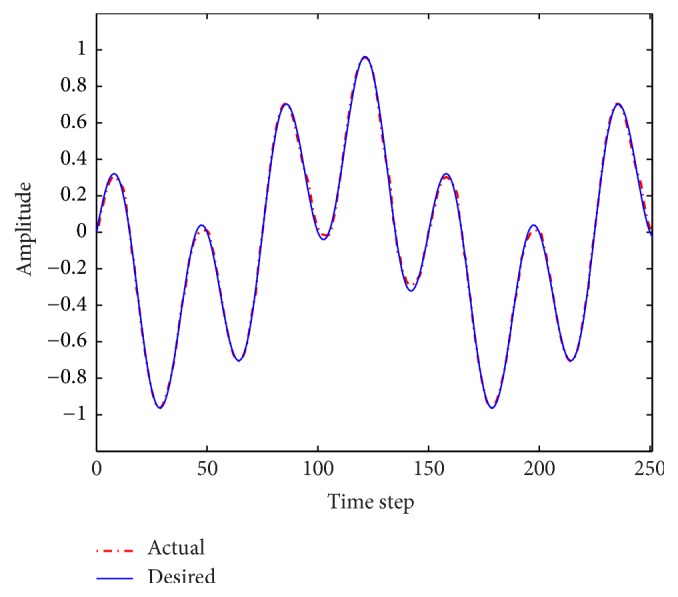
Fuzzy control results using ACODM-I in Example 1.

The performance results of the fuzzy system optimized by ACO_R_, ACODM, and ACO_R_-I (representing the ACO_R_ using the proposed initialization) are also provided in order to discuss or validate the effectiveness of the proposed initialization and dynamic mutation.  Table 3 presents these learned statistical numerical results when the value of *q* is 0.1. Simulation results show that ACODM achieved the smaller average error than ACO_R_. The average error of ACODM-I is also smaller than that of the ACO_R_-I. This indicates that the dynamic mutation can improve the optimization accuracy thus validating the effectiveness of the introduced dynamic mutations. The main reason to this is the ability of the dynamic mutation providing more diverse search directions for the ant colony when the value of *q* is not large. Similarly, the effectiveness of the proposed initialization on improving the accuracy of the fuzzy system design is also validated by the observation that the average errors of ACO_R_-I and ACODM-I are smaller than the ones of ACO_R_ and ACODM, respectively. Moreover, the learning curves of ACO_R_, ACODM, ACO_R_-I, and ACODM-I are shown in Figure 3. It is also observed that the algorithms incorporating dynamic mutation, ACODM, and ACODM-I converged slower than the ones without incorporating dynamic mutation, ACO_R_, and ACO_R_-I, respectively. The possibly best explanation is that the exploration on more search directions by the dynamic mutation brings the accuracy improvement at the expense of the convergence rate. Finally, ACODM-I achieved the smallest error. The tracking results controlled by ACODM-I optimized fuzzy system are presented in Figure 4. The results show that the controlled plant output is very close to the reference trajectory.

**Example 2 .**

The nonlinear plant for control using a zero-order TSK-type fuzzy system as taken in [15, 25] is described by

(18) $$\begin{array}{c} y\left(\;k+1\;\right)=\frac{y\left(k\right)y\left(k-1\right)\left(y\left(k\right)+2.5\right)}{1+y^{2}\left(k\right)+y^{2}\left(k-1\right)}+u\left(\;k\;\right). \end{array}$$

The initial states *y*(−1) and *y*(0) are assumed to −6, and −1.2 ≤ *u*(*k*) ≤ 1.2 is the control input of the plant. The objective of the zero-order TSK-type fuzzy controller is to control the plant output *y*(*k*) to track the reference trajectory 0.2*y*_*d*_(*k*), where *y*_*d*_(*k*) is governed by the reference model

(19) ydk+1=0.6ydk+0.2ydk−1+rk,0≤k<250,rk=0.2sin⁡2kπ25+0.4sin⁡kπ32,

where the initial states of the reference model *y*_*d*_(−1) = *y*_*d*_(0) = −6 are assumed. Two previous system outputs *y*(*k*), *y*(*k* − 1) and the target output 0.2*y*_*d*_(*k* + 1) are the three input variables of the fuzzy controller. The produced output *u*(*k*) of the fuzzy system is to control the nonlinear plant (18). The performance evaluation of a designed controller is defined as the sum of absolute error (SAE) between the plant output and reference trajectory over 250 time steps and is computed by

(20) $$\begin{array}{c} SAE=\overset{250}{\underset{k=1}{\sum }}\left|\;0.2y_{d}\left(\;k\;\right)-y\left(\;k\;\right)\;\right|. \end{array}$$

**Figure 5:**
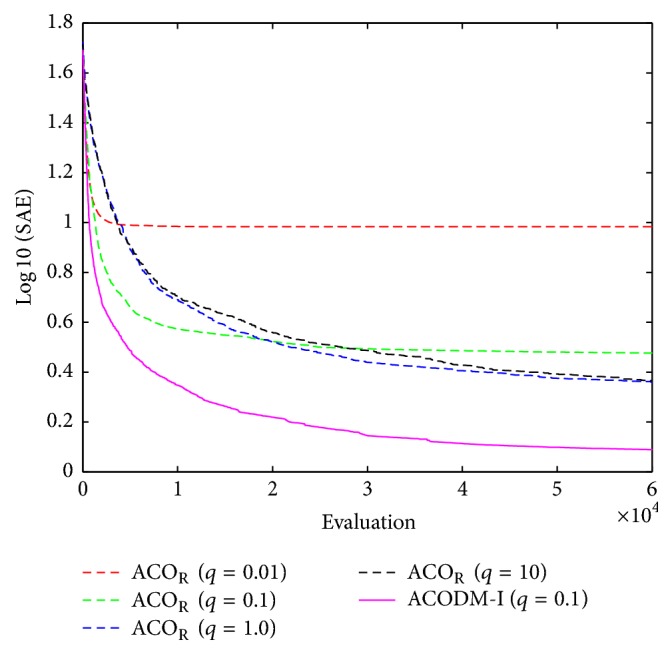
The average best-so-far SAE at each performance evaluation for the evolutionary fuzzy controllers optimized by ACODM-I and ACO_R_ in Example 2.

The fuzzy system to be optimized consists of four fuzzy rules. The values of the free parameters *m*_*ij*_ ∈ [−1.2,1.2], *b*_*ij*_ ∈ [0,1.2], and *a*_*i*_ ∈ [−1.2,1.2] are searched to find a better-performed fuzzy controller yielding smaller SAE. The 60000 evaluations were performed to conclude a solution in each run of optimization process. Over 50 runs of simulations, the average best-so-far SAE at each performance evaluation of each algorithm for fuzzy system design is shown in Figure 5. The learned numerical results of SAE errors are shown in Table 4. The results show that ACODM-I achieved the smaller error than ACO_R_ algorithms with different values of* q*.

ACODM-I performance is also compared to the reported results of HPSO-TVAC, HGAPSO, PSO-CREV, RCACO, and ant and particle swarm cooperative optimization (APSCO) using the parallel combination of ACO and PSO [15] when they were applied to the same fuzzy control problem. The comparison is shown in Table 5. The results show that ACODM-I achieved smaller average error than all other algorithms in comparison.

**Figure 6:**
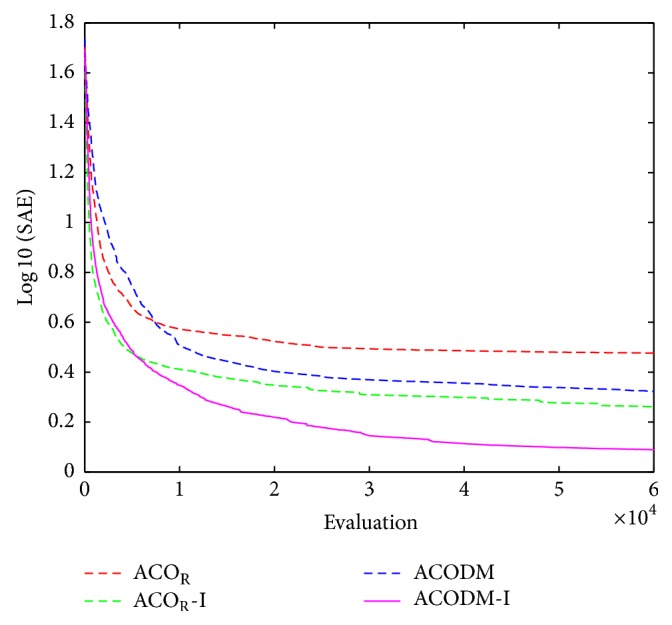
The average best-so-far SAE at each performance evaluation for the evolutionary fuzzy controllers optimized by ACO_R_, ACODM, ACO_R_-I, and ACODM-I in Example 2.

**Figure 7:**
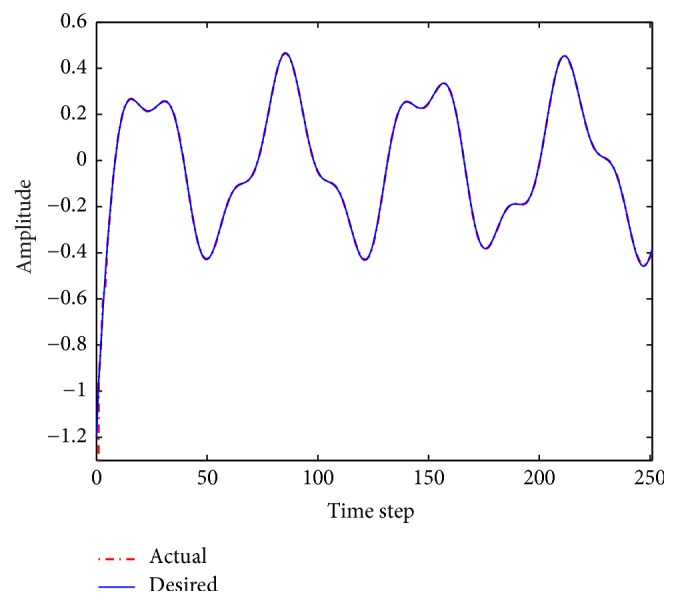
Fuzzy control results using ACODM-I in Example 2.

In this example, when the value of* q* is 0.1, simulation results of the zero-order TSK-type fuzzy controller optimized by ACO_R_, ACODM, ACO_R_-I, and ACODM-I are shown in Table 6 and Figure 6. In a similar way to Example 1, the effects of the proposed initialization and dynamic mutation on the design accuracy and convergence rate are easily identified in the simulation results. The tracking results of the fuzzy system optimized by ACODM-I are shown in Figure 7. The simulation results show the controlled plant output is very close to the desired reference trajectory.

**Example 3 .**

In this example, a first-order TSK-type fuzzy system is used to predict the chaotic time series. The time series to be predicted is generated by the well-known Mackey-Glass chaotic system, which is defined by the following time-delay differential equation

(21) $$\begin{array}{c} \frac{dx\left(t\right)}{dt}=\frac{0.2x\left(t-\tau \right)}{1+x^{10}\left(t-\tau \right)}-0.1x\left(\;t\;\right), \end{array}$$

where *τ* is set as 17, the initial point *x*(0) is assumed to be 1.2, and *x*(*t*) = 0 for *t* < 0 as in the previous studies [26, 27]. Four past values of *x*(*t* − 24), *x*(*t* − 18), *x*(*t* − 12), and *x*(*t* − 6) are the inputs of the first-order TSK-type fuzzy system. The produced output of the fuzzy system excited by these four past inputs is the prediction of *x*(*t*). The fuzzy predictor is optimized by ACODM-I and evaluated in the following experiment. First, the solution of (21) was solved using Runge-Kutta numerical method. Secondly, the time series data were generated by sampling the solution every second. The sampled data patterns from *t* = 124 to 623 were used as the training set for optimizing the fuzzy system while the remaining patterns from *t* = 624 to 1123 were used as test set for validating the designed fuzzy model.

**Figure 8:**
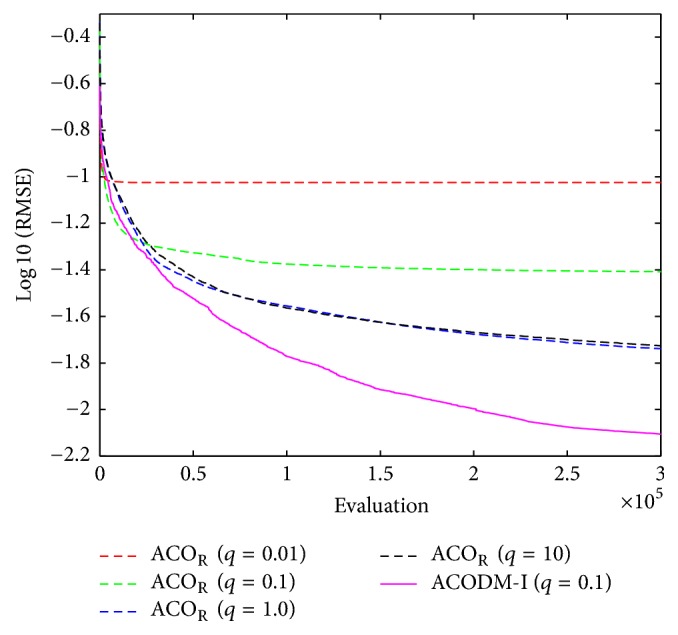
The average best-so-far RMSE at each performance evaluation for the evolutionary fuzzy predictor optimized by ACODM-I and ACO_R_ in Example 3.

The first-order TSK-type fuzzy system consists of four rules. The values of the free parameters *m*_*ij*_ ∈ [0,2], *b*_*ij*_ ∈ [0,1], and *a*_*ij*_ ∈ [−2,2] are searched to find a better-performed fuzzy predictor. The objective function is defined as the RMSE between the sampled output of the chaotic system governed by (21) and the predicted output by the fuzzy system. The 300000 evaluations were performed in a single run of training. The learning results of the average RMSE at each evaluation over 50 runs are shown in Figure 8.  Table 7 shows the learned numerical results of training and testing RMSE errors. The results show that ACODM-I achieved the smaller prediction error than the ACO_R_ algorithms with different values of *q*.

**Figure 9:**
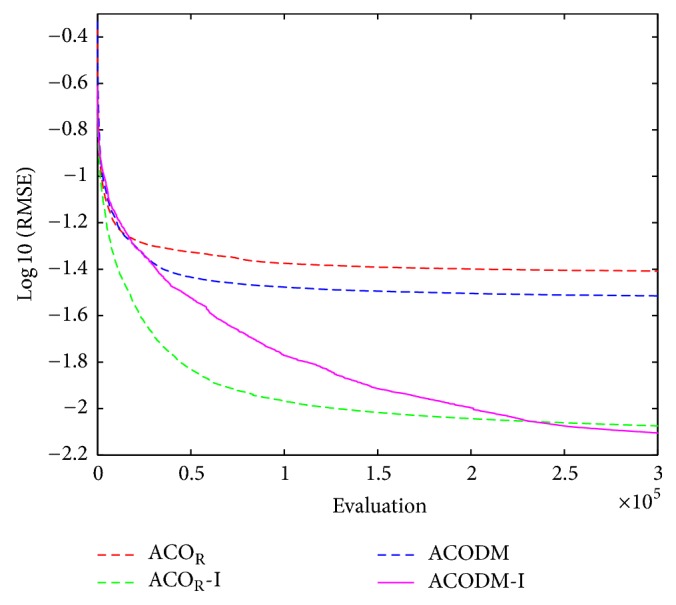
The average best-so-far RMSE at each performance evaluation for the evolutionary fuzzy predictor optimized by ACO_R_, ACODM, ACO_R_-I, and ACODM-I in Example 3.

**Figure 10:**
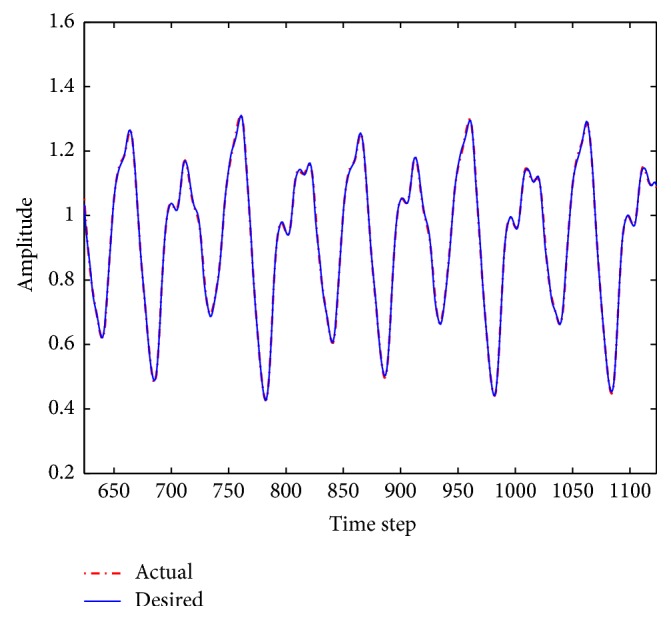
Prediction results of the fuzzy system optimized by ACODM-I in Example 3.

Similar to the two previous examples, simulation results of the first-order TSK-type fuzzy systems optimized by ACO_R_, ACODM, ACO_R_-I, and ACODM-I are provided in Table 8 and Figure 9 to verify the effects of the proposed initialization and dynamic mutation. The results indicate that the proposed initialization and dynamic mutation in ACODM-I as applied to the design of first-order TSK-type fuzzy system have the same effects as those used in the zero-order TSK-type fuzzy systems for control problems. It is also observed that the proposed initialization helps improve much more on design accuracy than dynamic mutation. The predication results of the first-order TSK-type fuzzy system optimized by ACODM-I are shown in Figure 10. The results show the prediction values by the fuzzy system are very close to the desired ones.

The performance of fuzzy system designed by ACODM-I is also compared to the reported results of different algorithm and neural-fuzzy systems that were applied to the same prediction problem [28]. These neural-fuzzy systems include a Hybrid Neural-Fuzzy Inference System (HyFIS) [35], a Dynamic Fuzzy Neural Network (D-FNN) [36], a Subsethood-Product Fuzzy Neural Inference System (SuPFuNIS) [37], and a Generalized Fuzzy Neural Network (G-FNN) [38]. The algorithm in comparison is a multiple-colony topology based Cooperative Continuous ACO- (CCACO-) designed neural-fuzzy system [26]. The comparison results are shown in Table 9. The results show the fuzzy predictor optimized by ACODM-I achieved the smaller test error than the most of reported neural-fuzzy systems. Though the ACODM-I performance is very close to the G-FNN and CCACO, ACODM-I optimized fuzzy system uses smaller number of rules than G-FNN. As reported in [26], the average training RMSE of the CCACO is 0.0094 which is larger than 0.0078 of ACODM-I. Moreover, the CCACO algorithm is a multiple-colony algorithm, whose computation is more complex than the simple single-colony algorithm such as ACODM-I.

## 5. Conclusions

This paper proposes an enhanced ant colony optimization with dynamic mutation and ad hoc initialization, ACODM-I, for improving the accuracy of TSK-type fuzzy systems design. ACODM-I is developed based on ACO_R_ and is regarded as a new population-based evolutionary optimization algorithm. Based on the observations of some accuracy-oriented fuzzy controllers, this paper proposes an ad hoc population initialization for initial ant solutions to improve the design accuracy. This is an application-specific initialization rather than generic initialization currently used in most population-based algorithms. ACODM-I incorporates the dynamic mutation technique into ACO_R_ to balance exploration ability and convergence rate. In addition to exploiting local search around the chosen leading solution using Gaussian sampling, the dynamic mutation probabilistically provides more search directions by “jumping” to the neighboring of other archive solutions in order to avoid being trapped into a local optimum.

To validate the proposed algorithm, three application examples of the TSK-type fuzzy system designs have been simulated: two zero-order TSK-type fuzzy systems for nonlinear dynamic plant control and one first-order TSK-type fuzzy system for the prediction of the chaotic time series. The simulation results have demonstrated that ACODM-I achieves smaller error than the ones by the ACO_R_ algorithms with different values of* q. *The effects on the design accuracy and convergence rate yielded by the proposed initialization and introduced dynamic mutation have also been discussed and verified in the simulations. In addition, the comparison with some advanced population-based algorithms also shows that the error achieved by ACODM-I is smaller than those by those algorithms used for comparison including HPSO-TVAC, PSO-CREV, HGAPSO, TPSIA, APSCO, and RCACO for the design of zero-order TSK-type fuzzy controller and CCACO for the design of first-order TSK-type fuzzy predictor.

In the future, the performances of ACODM-I for the designs of various types of feed-forward or recurrent fuzzy systems will be studied. The adaptive mechanism or algorithm of adjusting the value of *h* in (14) determining the mutation probability will be investigated in the future study. The hybridization of ACODM-I with other computational techniques may further improve optimization accuracy; thus it is possibly worthy of being studied in the future.

## Figures and Tables

**Algorithm 1:**
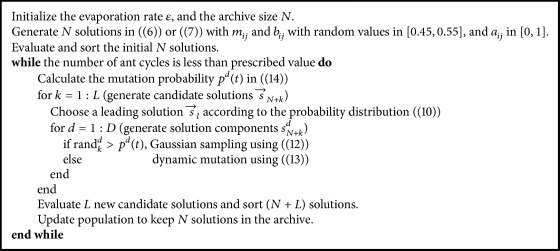
ACODM-I algorithm for TSK-type fuzzy system design.

**Table 1:** Performances of the fuzzy controller designed by the ACODM-I and ACO_R_ in Example 1.

Algorithms	ACO_R_ (*q* = 0.01)	ACO_R_ (*q* = 0.1)	ACO_R_ (*q* = 0.2)	ACO_R_ (*q* = 0.4)	ACO_R_ (*q* = 0.6)	ACO_R_ (*q* = 0.8)	ACO_R_ (*q* = 1.0)	ACO_R_ (*q* = 10)	ACODM-I (*q* = 0.1)
Average RMSE	0.0525	0.0350	0.0343	0.0357	0.0354	0.0392	0.0374	0.0403	**0.0256**
STD	0.0241	0.0071	0.0067	0.0057	0.0049	0.0066	0.0056	0.0050	0.0032
Minimum	0.0273	0.0220	0.0231	0.0261	0.0269	0.0300	0.0280	0.0276	**0.0201**
Maximum	0.1835	0.0524	0.0491	0.0472	0.0454	0.0575	0.0490	0.0518	**0.0372**

**Table 2:** Performances of fuzzy controller designed by the ACODM-I and different algorithms in Example 1.

Algorithms	HPSO-TVAC	HGAPSO	PSO-CREV	TPSIA	RCACO	ACODM-I
Average RMSE	0.039	0.040	0.041	0.033	0.0260	**0.0256**
STD	0.014	0.008	0.012	0.012	0.0047	0.0032

**Table 3:** Performances of fuzzy controller designed by ACO_R_, ACODM, ACO_R_-I, and ACODM-I in Example 1.

Algorithms	ACO_R_	ACO_R_-I	ACODM	ACODM-I
Average RMSE	0.0350	0.0275	0.0299	**0.0256**
STD	0.0071	0.0053	0.0061	0.0032
Minimum	0.0220	0.0215	0.0217	**0.0201**
Maximum	0.0524	0.0441	0.0433	**0.0372**

**Table 4:** Performances of the fuzzy controller designed by ACODM-I and ACO_R_ in Example 2.

Algorithms	ACO_R_ (*q* = 0.01)	ACO_R_ (*q* = 0.1)	ACO_R_ (*q* = 0.2)	ACO_R_ (*q* = 0.4)	ACO_R_ (*q* = 0.6)	ACO_R_ (*q* = 0.8)	ACO_R_ (*q* = 1.0)	ACO_R_ (*q* = 10)	ACODM-I (*q* = 0.1)
Average SAE	9.6332	2.9983	2.3056	2.5632	2.1049	2.4515	2.2922	2.3184	**1.2292**
STD	8.6435	2.6633	1.7033	1.6424	0.8539	1.5094	0.8167	1.3302	0.7454
Minimum	1.6844	0.722	0.5101	0.7012	0.838	0.9103	0.686	0.9544	**0.4879**
Maximum	58.4138	13.0973	12.1271	9.7500	3.7552	9.9463	3.4403	9.0152	**2.8749**

**Table 5:** Performances of fuzzy controller designed by the ACODM-I and different algorithms in Example 2.

Algorithms	HPSO-TVAC	HGAPSO	PSO-CREV	APSCO	RCACO	ACODM-I
Average SAE	6.64	5.33	4.0	3.73	1.92	**1.23**
STD	5.64	2.89	1.2	1.65	1.06	0.75

**Table 6:** Performances of fuzzy controllers designed by ACO_R_, ACODM, ACO_R_-I, and ACODM-I in Example 2.

Algorithms	ACO_R_	ACO_R_-I	ACODM	ACODM-I
Average SAE	2.9983	1.8273	2.1049	**1.2292**
STD	2.6633	0.9141	0.879	0.7454
Minimum	0.722	0.5465	0.5174	**0.4879**
Maximum	13.0973	3.2088	4.179	**2.8749**

**Table 7:** Performances of the fuzzy predictor designed by the ACODM-I and ACO_R_ in Example 3.

Algorithms	ACO_R_ (*q* = 0.01)	ACO_R_ (*q* = 0.1)	ACO_R_ (*q* = 0.2)	ACO_R_ (*q* = 0.4)	ACO_R_ (*q* = 0.6)	ACO_R_ (*q* = 0.8)	ACO_R_ (*q* = 1.0)	ACO_R_ (*q* = 10)	ACODM-I (*q* = 0.1)
Training Average	0.0944	0.0391	0.0364	0.0173	0.0187	0.0184	0.0183	0.0188	**0.0078**
Test Average	0.0934	0.0386	0.0359	0.0171	0.0184	0.0182	0.0180	0.0186	**0.0078**
Test STD	0.0262	0.035	0.0347	0.0067	0.0044	0.0039	0.0037	0.0039	0.0012
Test Minimum	0.0364	0.0087	0.0102	0.0079	0.0095	0.0123	0.0097	0.0113	**0.0058**
Test Maximum	0.1553	0.0962	0.0962	0.0532	0.0337	0.0266	0.0294	0.0281	**0.0119**

**Table 8:** Performances of fuzzy predictor designed by ACO_R_, ACODM, ACO_R_-I, and ACODM-I in Example 3.

Algorithms	ACO_R_	ACO_R_-I	ACODM	ACODM-I
Test Average	0.0386	0.0084	0.0303	**0.0078**
Test STD	0.0350	0.0012	0.0314	0.0012
Test Minimum	0.0087	**0.0057**	0.0079	0.0058
Test Maximum	0.0962	0.0131	0.0962	**0.0119**

**Table 9:** Performances of ACODM-I, CCACO, and different neural-fuzzy systems in Example 3.

Models	HyFIS	D-FNN	SuPFuNIS	G-FNN	CCACO	ACODM-I
Rule number	16	5	4	10	4	4
Test RMSE	0.0100	0.0131	0.0075	**0.0056**	0.0061	0.0058
